# Engineering NiO/g-C₃N₄ and NiO/rGO composites for dual applications in electrochemical water splitting and energy storage

**DOI:** 10.1038/s41598-025-20713-3

**Published:** 2025-10-21

**Authors:** M. Manikandan, E. Anandharam, E. Manikandan, Saheli Karmakar

**Affiliations:** 1 School of Electronics Engineering, Vellore Institute of Technology, Chennai, 600127 India; 2 School of advance Science, Vellore Institute of Technology, Chennai, 600127 India; 3 Centre for Advanced Materials and Innovative Technologies, Vellore Institute of Technology, Chennai, 600127 India

**Keywords:** NiO/rGO, NiO/g-C₃N₄, Supercapacitor, Metal oxide composite, Electrochemical water splitting, HER, Energy density and power density, Chemistry, Energy science and technology, Materials science, Nanoscience and technology

## Abstract

The development of multifunctional electrode materials that can simultaneously serve as efficient electrocatalysts and high-performance energy storage devices is highly desirable for next‐generation energy conversion technologies. In this work, we report a facile hydrothermal strategy followed by annealing at 400 °C, which enables the in-situ reduction of GO to rGO without external reducing agents, leading to robust NiO/rGO and NiO/g-C₃N₄ nanocomposites. This approach ensures intimate interfacial contact between NiO and the conductive carbon matrix, thereby enhancing charge transfer and catalytic activity. The composites were systematically characterized using XRD, BET, Raman, FESEM–EDX, XPS, and TEM analyses to confirm structural and surface features. Electrochemical evaluation revealed that NiO/rGO and NiO/g-C₃N₄ electrodes delivered low overpotentials of 126 and 73 mV at 10 mA cm⁻² for HER in 1 M KOH, with NiO/g-C₃N₄ exhibiting long-term stability over 23 h. As supercapacitor electrodes, NiO/rGO and NiO/g-C₃N₄ achieved remarkable specific capacitances of 597 and 366 F g⁻¹ at 1 A g⁻¹, respectively, surpassing many previously reported NiO–carbon systems. These results demonstrate that the unique synthesis route and synergistic coupling of NiO with conductive carbon frameworks enable a substantial advancement in multifunctional electrode design, offering a promising pathway for integrated energy conversion and storage systems.

## Introduction

Since, the global demand for sustainable energy grows, the search for eco-friendly and an efficient energy conversion and storage solutions has become more crucial than ever. Among several approaches, electrochemical water splitting and supercapacitors have emerged as promising technologies to address energy challenges^1–3^. Water splitting, particularly the hydrogen evolution reaction (HER), presents a clean and renewable technique for producing hydrogen fuel, a key candidate for replacing fossil fuels^4,5^. Simultaneously, the advancement of electrochemical energy storage devices, such as supercapacitors, is crucial for fulfilling the energy demands of modern electronic and power grid systems. On the other hand, due to their high-power energy storage with rapid charge/discharge cycles and long lifespan make them essential for next-generation energy storage systems^6,7^.

In contrast to conventional batteries, a supercapacitor is a sophisticated energy storage device that stores energy either by quick surface redox reactions (in pseudo capacitors) or electrostatic interactions (in electric double-layer capacitors, EDLCs). Compared to batteries, supercapacitors offer higher power density, longer cycle life, and faster energy delivery, making them attractive for applications in electric vehicles, portable electronics, and grid energy storage^8,9^. Concurrently, in electrochemical water splitting, the HER plays a crucial role by producing hydrogen at the cathode via a series of electron transfer reactions. However, HER is often hindered by slow reaction kinetics and high overpotentials, necessitating the development of highly efficient and cost-effective catalysts^10,11^.

Among various materials explored for HER and supercapacitor applications, transition metal oxides (TMOs) have garnered significant recognition due to their earth abundance, structural tunability, and rich redox active site. Several TMOs, including Co₃O₄, MnO₂, MoO₃, CuO, Fe₂O₃, and NiO, have been extensively studied for their dual applicability in energy conversion and storage systems^12,13^. Electrophoretic self-assembled nano-Co_3_O_4_ films was synthetized by Guo et al. where they reported the specific capacitance of 233.6 F g^-1^ at a current density of 0.5 A g^-1^^14^. Sohouli et al. used a flower like nanocomposite of CuO/NCNO as a high performance supercapacitor electrode, which delivers 455 F g^-1^ specific capacitance and high steadiness after 3000 cycles^15^. Wang et al. successfully synthesized δ-MnO_2_ nanosheets from enteromorpha prolifera (ACEP) which exhibits high specific capacitance of 345.1 F g^−1^ at 0.5 A g^−1^ and high cycle stability with 92.8% retention of capacitance after 5000 cycles^16^. Liu et al. reported Mn_3_O_4_ nanodots with Nitrogen doped graphite material with a specific conductance of 158.9 F g ^-1^^17^. Y Wang et al. fabricated Poly-pyrrole-encapsulated Fe_2_O_3_ nanocomposite arrays which display 237 mF cm^-2^ capacitance at current density of 1 mA cm^-2^^18^. Jeon et al. prepared RuO_2_ nanorods on electrospun carbon nanofiber which delivers a specific capacitance of 188 F g^-1^ at current density 1 mA cm^-2^^19^. A cost-effective material with promising electrochemical properties but suffers from lower capacitance and catalytic activity compared to other TMOs^20^.

Out of all TMOs, nickel oxide (NiO) stands out for affordability natural abundance, excellent electrochemical stability, and well-defined redox activity (Ni²⁺/Ni³⁺ transitions)^21,22^. In HER applications, NiO can serve as an efficient electrocatalyst in alkaline media, particularly when coupled with conductive and catalytic supports. By interface engineering, a Zn-dopped NiO heterostructure was created by Kiran et al. which exhibit overall water splitting with overpotential of 88mV at 10 mA cm^-2^ for hydrogen evolution reactions (HER) 260 mV at 10 mA cm^-2^for oxygen evolution reaction (OER)^23^. A phosphorous and aluminum-coated porous NiO nanosheet that is an effective electrocatalyst for overall water splitting was presented by Li et al. In 1 M KOH, it can support a current density of 100 mA cm^-2^ for the oxygen evolution reaction (OER) at an overpotential of 310 mV and for the hydrogen evolution reaction (HER) at an overpotential of 138 mV. ^24^ Goel et al. developed NiO nanoflakes by microwave assisted synthesis method. This NiO nanoflakes act as pseudo supercapacitor with specific capacitance of 307 F g at 0.5 A g^-1^.^25^ Han and co-worker used Mn dopped NiO nanosheet arrays to enhance capacitive and they achieved high specific capacitance of 1166 F g^-1^ at the current density of 5 mA cm^-2^^26^. Kwek et al. fabricated NiO embedded, nanofiber composite electrode having specific capacitance of 161 F g^-1^ at 1 A g^-1^^27^. Tong et al. developed a heterojunctions between, N-doped amorphous carbon, Ni and NiO quantum dots to obtain high specific capacitance of 223.2 F g^-1^ at a high current density of 20 A g^-1^^28^. However, the key challenge with NiO is its low intrinsic conductivity (~ 10^−13^S/cm), which hinders its charge transfer kinetics in both supercapacitors and HER applications^29^.

To mitigate the conductivity limitations of NiO and improve its electrochemical activity, composite structures with carbon-based materials have been explored. In this study, we utilize reduced graphene oxide (rGO) and graphitic carbon nitride (GCN**)** to engineer NiO-based hybrid materials for enhanced energy storage and water splitting performance. rGO is known for its superior charge transport capability, large surface area, and high electrical conductivity, making it ideal for enhancing the electrochemical performance of NiO. On the other hand, GCN, a nitrogen-rich 2D material, provides additional active sites, enhances charge storing capacity, and improves the durability of the composite through strong interactions with NiO^30,31^.

**Fig. 1:**
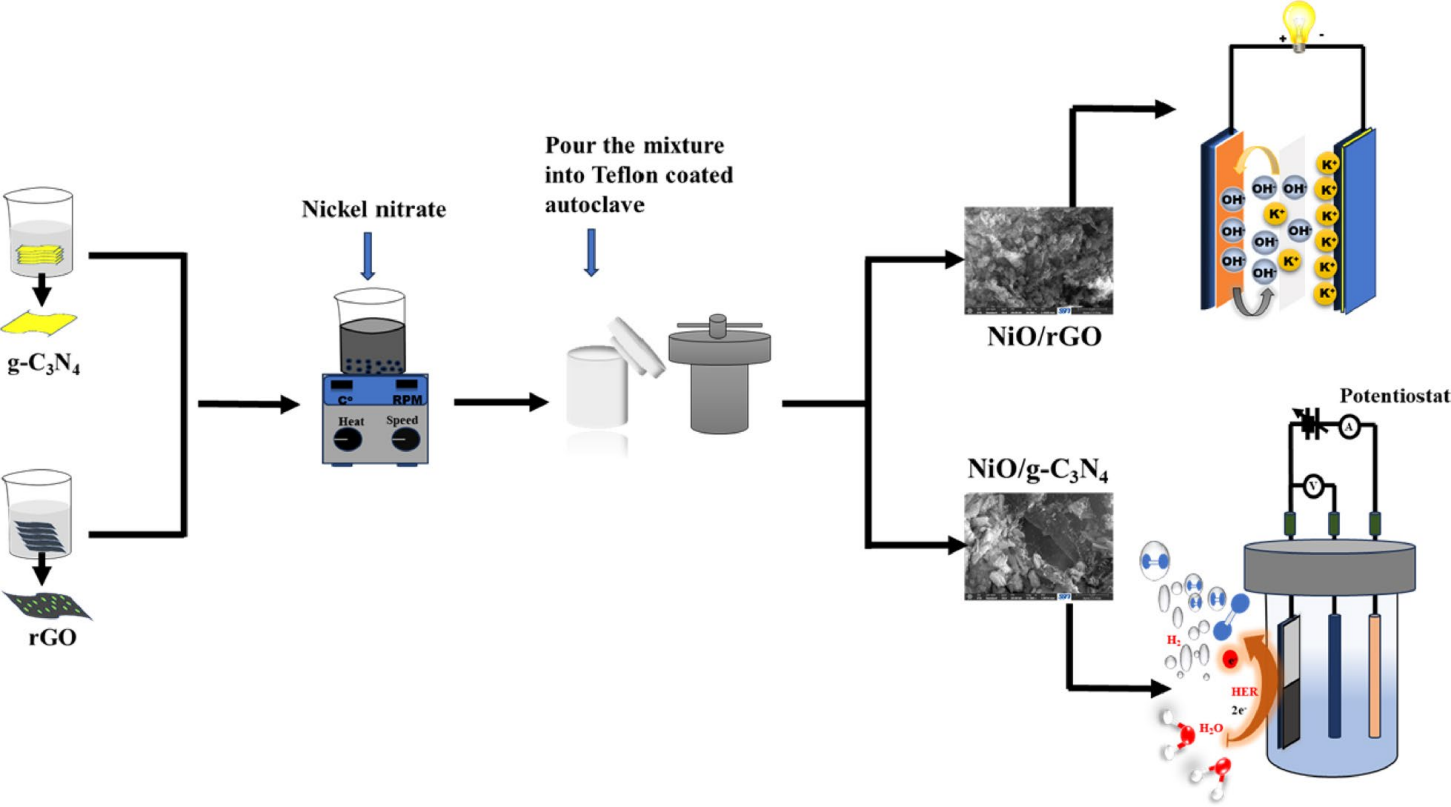
Synthesis of NiO/rGO and NiO/g-C₃N₄ composites.

In this work, we have synthesised NiO/rGO and NiO/g-C₃N₄ composites by a simple hydrothermal method. These composite exhibits an excellent electrochemical features with dual application in energy storage as supercapacitor and energy conversion through water splitting. The synthesized composites demonstrate capacitance of 597 F g^−1^ and 366 F g^−1^ at current density of 1 A g^−1^ for NiO/rGO and NiO/g-C₃N₄ respectively. They shows the competence capacitance stability (maintaining 97% and 96% after 5000 cycles, correspondingly) and the constructed supercapacitor device with NiO/rGO yields remarkable energy and power densities of 36.88 W h kg^−1^ and 1599.61 W kg^−1^, with specific capacities of 103 F g^−1^ at current density 1 A g^−1^.Moreover, over potential of 126 mV and 73 mV (HER) has been delivered by the NiO/rGO and NiO/g-C₃N₄ composite respectively. The synthesis methods of NiO/rGO and NiO/g-C₃N₄ composite is illustrated in Fig. 1.

## Experimental section

### Materials

Nickel(II) Nitrate Hexahydrate (Ni(NO3)2.6H2O) were purchased in Sisco research laboratories Pvt. Ltd, Graphene oxide (GO) were purchased in ADNANO technologies Pvt. Ltd, Potassium hydroxide used as a electrolyte and distilled water.

### Synthesis of g-C₃N₄

Melamine was used as the precursor in a thermal polymerization process to create graphitic carbon nitride (g-C₃N₄). A covered alumina crucible containing 5 g of melamine was heated in a muffle furnace as part of a standard synthesis. Under ambient atmospheric conditions, the sample was heated to 550 °C at a rate of 5 °C min⁻¹ and kept there for four hours. The heating process facilitated the polymerization and condensation of melamine into g-C₃N₄. The furnace was left to naturally cool to room temperature following the conclusion of the thermal treatment. To obtain g-C₃N₄, the resultant pale yellow solid was gathered and pounded into a fine powder using a granite mortar and pestle.

### Synthesis of NiO/g-C₃N₄

A facile hydrothermal method is used to create NiO/g-C₃N₄ composite. Using a standard method, 1.5 gm Ni(NO_3_)_2_.6H_2_O, was dissolved in 40 ml of deionized water while being stirred magnetically. 1.5 gm of g-C₃N₄ was then added to the solution, which was left at room temperature for 30 min. The solvent was transferred in a 100 ml Teflon lined, stainless steel autoclave and heated to 120 °C for 15 h. The generated sample was repeatedly cleaned in ethanol and deionized (DI) water to get rid of any last bits of unreacted material once the autoclave had cooled to room temperature. It was then dried in a vacuum oven nightly at 80 °C. Finally dried sample was kept for calcination in muffle furnace at 400 °C for 2 h.

### Synthesis of NiO/rGO

To synthesize NiO/rGO, 40 mg of Graphene Oxide was suspended in a 40 ml of DI water and sonicated for 2 h. Subsequently, 1.5 gm of nickel nitrate hexahydrate, was put on to the solution, while stirring it with a magnetic ^-1^stirrer. Then remaining procedure was carried out again which is similar with NiO/g-C₃N₄. The subsequent steps, which are comparable with NiO/g-C₃N₄, were carried out again^32^.

## Electrochemical cell

In our supercapacitor measurements, a three-electrode configuration was employed. A platinum wire was used as the counter electrode, an Ag/AgCl (saturated KCl) electrode served as the reference electrode, and the active-material-coated Ni foam was used as the working electrode. All measurements were performed in 3 M of KOH aqueous electrolyte at room temperature. The specific capacity of the electrode materials was calculated from the following formula^5,8^:

1 $$C{\text{ }} = {\text{ }}I\Delta t/m\Delta v$$

where *C* is the specific capacity of the electrode materials (F g^−1^), *I* is the discharge current (A g^−1^), Δ*t* is the discharge time (s), and *m* is the mass of the active material (g).

Where *q*_+_ and *q*_−_ are the stored charges in the positive and negative electrode surfaces, respectively. The amount of charge stored on the electrodes depends on the capacity (*C*) and mass of the electrode materials (*m*). Thus, the mass balance equation is expressed as follows^5,8^:

2 $$q^+ {\text{ }} = {\text{ }}C^+ {\text{ }} \times {\text{ }}m^+$$

3 $$q^- {\text{ }} = {\text{ }}C^- {\text{ }} \times {\text{ }}m^-$$

4 $$m^+ /m^- {\text{ }} = {\text{ }}C^- /C^+$$

The energy and power density values were calculated by the following Eqs^5,8^. :

5 $$E{\text{ }} = {\text{ }}CV2{\text{ }}/7.2$$

6 $$P{\text{ }} = {\text{ }}E{\text{ }} \times {\text{ }}3600/t$$

where the energy density (*E*), power density (*P*), *C* is the specific capacitance (c g^−1^), Δ*V* is the potential window (V), and Δ*t* is the discharging time (s).

## Results and discussion

**Fig. 2:**
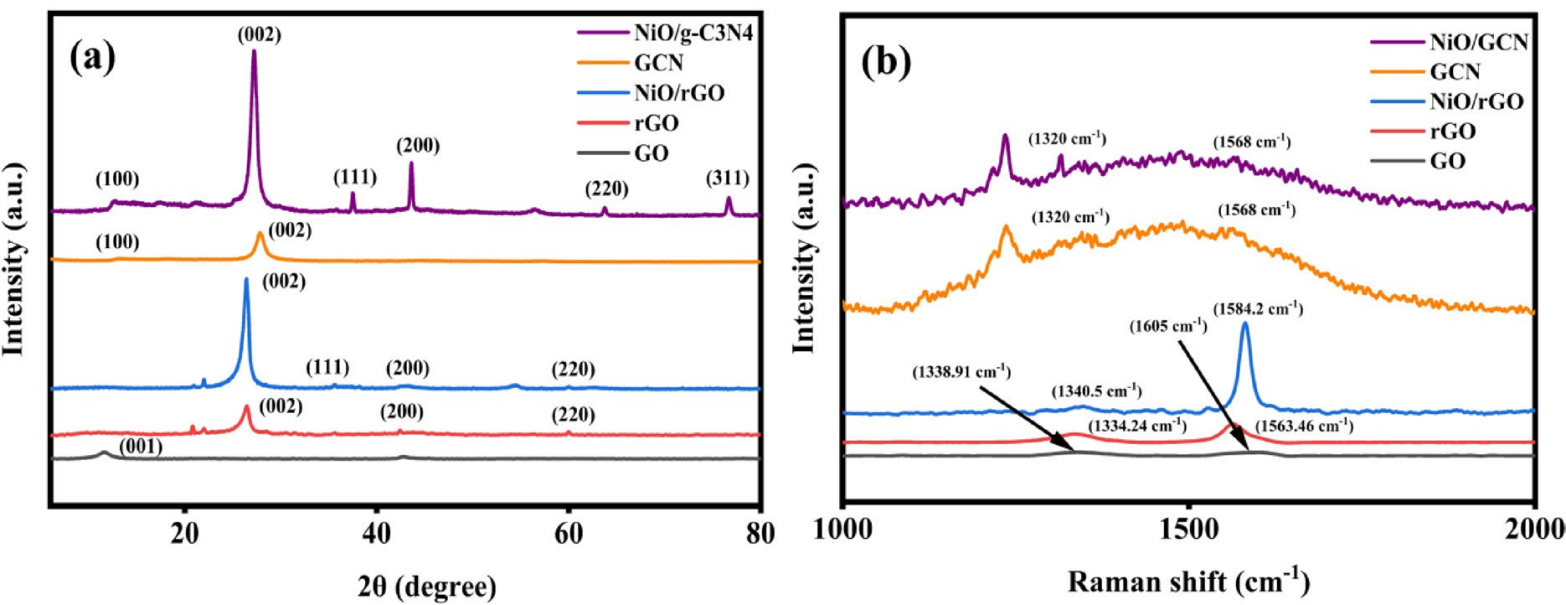
(**a**) XRD analysis and (**b**) Raman Spectra of NiO/g-C₃N₄ and NiO/rGO.

The powder X-ray diffraction (PXRD) patterns of GO, GCN, NiO/g-C₃N₄ and NiO/rGO nanocomposite has been displayed in Fig. 2a. The diffraction spectrum of NiO/g-C₃N₄ includes all of the characteristic diffraction peaks of tri-s-triazine-based g-C₃N The characteristic feature of the (002) crystal plane of g-C₃N₄ is the diffraction at 27.1° and the intra-layer d-spacing in g-C₃N₄ can be attributed to the diffraction at 12.9 °,which is characteristic of the (100) plane^33^. Furthermore, it exhibits prominent diffraction at 37.4°, 43.6°, 63.7° and 76.7° which attributes the characteristic diffraction of (111), (200), (220) and (311) crystal planes of cubic NiO respectively^33,34^. Whereas, NiO/rGO shows a prominent peak at 26.3° which is corresponds to (002) plans of bare rGO. In addition there are prominent peaks of NiO at 35.6°, 42.9°, 60.0° corresponding to the crystal planes of (111), (200), and (220) respectively^35^.

Figure 2b illustrates the Raman spectrum of GO, rGO, GCN, NiO/g-C₃N₄ and NiO@rGO nanocomposites. Graphene oxide attribution is provided the D and G bands at 1338.91 cm^−1^ and 1650 cm^−1^. Reduced graphene oxide(rGO) bands was peaked at 1334.24 cm^−1^ and 1568 cm^−1^.Graphitic carbon nitride given a number of bands seen in the 706.7 cm^−1^ to 1653.8 cm^−1^region of the Raman spectra of g-C₃N₄^33^. So, its shows the contribution in the composites. Moreover, the aromatic C–N heterocycles of the heptazine (melem) units, which are the fundamental building blocks of g-C₃N₄ is attributed to the stretching vibrations at 768.3 cm^−1^, 801 cm^−1^, 974.4 cm^−1^, 1112.8 cm^−1^, 1177.2 cm^−1^, 1235.7 cm^−1^, and 1315.7 cm^−1^.S-triazine’s several ring breathing modes are linked to the peaks at 706.7 cm^−1^ and 1000 cm^−1^. The presence of graphitic carbon nitrite is confirmed by the bands D (1320 cm^−1^) to G (1568 cm^−1^) in the Raman data. Similarly, the bands D (1340.5 cm^−1^) to G (1584.2 cm^−1^) in the Raman measurements demonstrate the presence of rGO^34,35^. The one phonon (1P) longitudinal optical (1P_LO_) and two phonons (2P) longitudinal optical (2P_LO_) of NiO vibrational modes are responsible for the two peaks seen in both spectra at 482.2 cm^−1^ and 995.2 cm^−1^, respectively^36^.

**Fig. 3:**
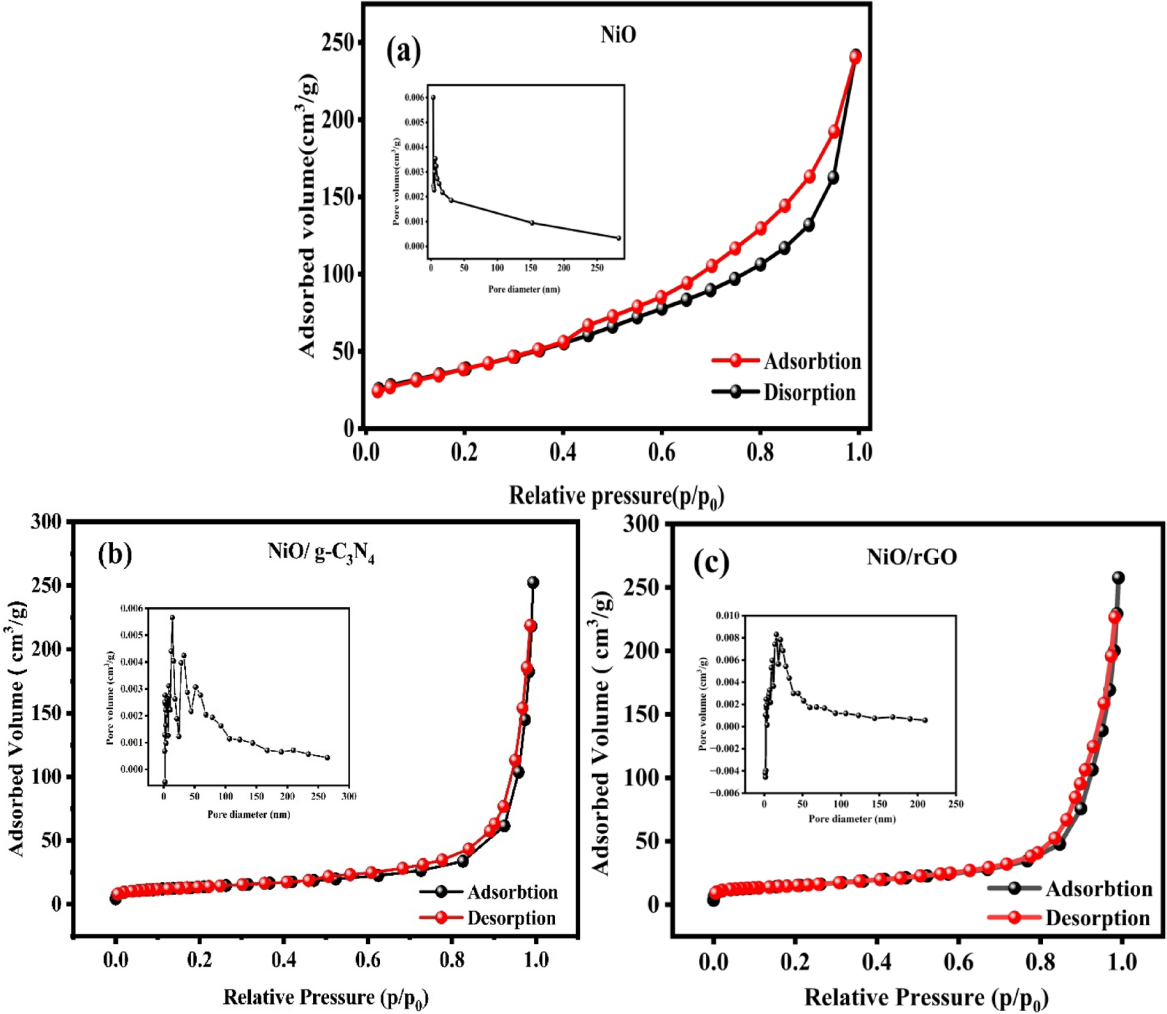
BET (**a**) NiO/g-C₃N₄ and (**b**) NiO/rGO composites (**c**) NiO bare about N2 adsorption–desorption isotherms with the pore diameter distribution in the insertion.

The nitrogen adsorption/desorption isotherms shown in the Fig. 3a exhibits the pore size distribution plots of hydrothermally synthesized NiO. The pore size of NiO were be calculated from the plot plot of NiO’s Bar-rett–Joyner–Halenda (BJH) Fig. 3 (a)displays. The hysteresis loop with high relative pressure (P/P0) ranges between 0 and 1, according to the NiO powder’s analytical isotherms. As seen in Fig. 3 (a), the slope increases from 0.2 to 0.4, indicating a mesoporous nature and high relative pressure. The plot indicates that the maximal BET surface area for NiO powder is 137.9 m2 g^−1^. In the porous region, the average pore radius of the NiO powder nanocluster structure is 3.41 nm^37^.

The BET analysis is shown in Fig. 3b, c to confirm the porous structure of NiO/g-C_3_N_4_ and NiO/rGO. For both the nanocomposite, the N_2_ adsorption-desorption isotherms displayed type-IV isotherms with an evident hysteresis loop at relative pressure P/P_0_ ranging from 0.7 to 1.0 and 0.8 to 1.0, respectively^38^. A typical distinction between adsorption and desorption on mesoporous materials is apparent by this curve. NiO/g-C_3_N_4_ and NiO/rGO have a pore volume of 0.3423 cm^3^/g, and 0.3856 cm^3^/g, a surface area of 46 m^2^ g^−1^, and 52 m^2^ g^−1^, and the average pore size of ~ 33.50 nm. and ~ 31.79 nm given by BJH model. By comparing with NiO/g-C_3_N_4_, NiO/rGO shows the high specific surface area and porous structure are beneficial to the adsorption and diffusion. ^38^

**Fig. 4:**
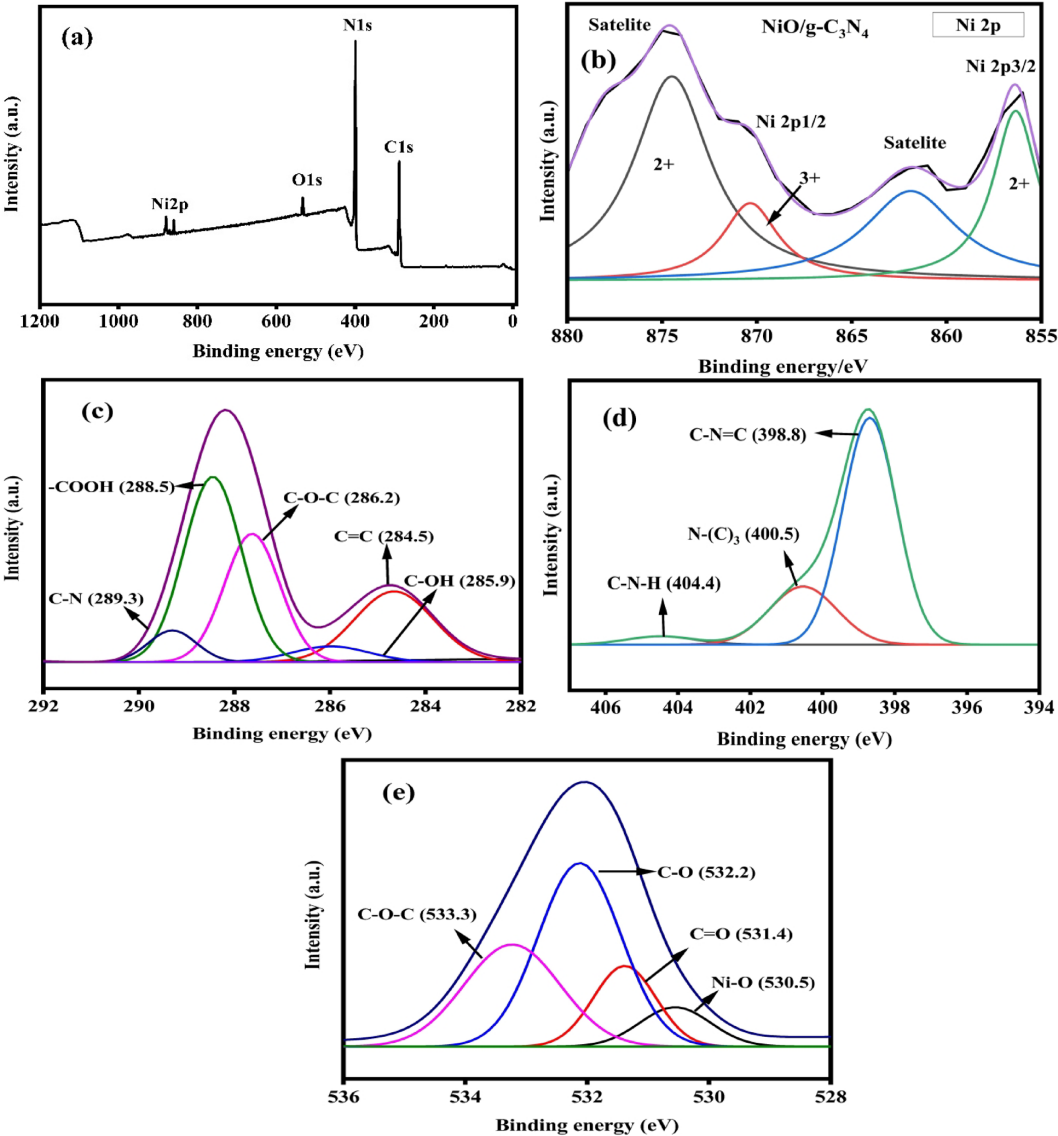
(**a**) XPS survey spectrum NiO/g-C3N4 and deconvoluted higher resolution XPS spectrum of (**b**)Ni 2p, (**c**) C 1 s, (**d**) N 1 s and (**e**) O 1s.

**Fig. 5:**
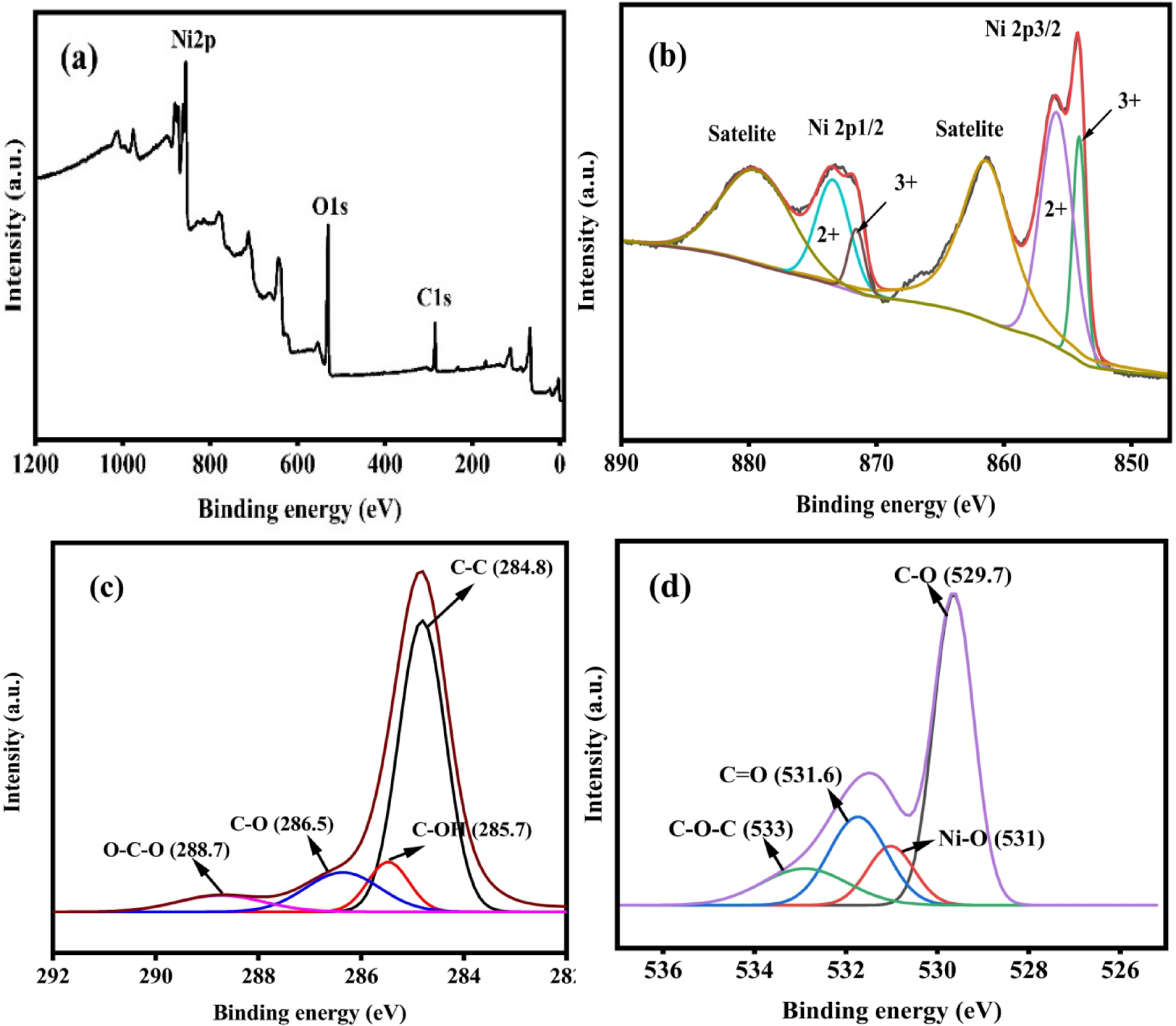
(**a**) XPS survey spectrum of NiO/rGO and deconvoluted higher resolution XPS spectrum of (**b**)Ni 2p, (**c**) C 1 s and (**d**) O 1s.

X-ray photoelectron spectroscopy (XPS) was performed to investigate the NiO/GCN and NiO/rGO composite’s oxidation state and detailed elemental information. Figure 4a shows the presence of nickel, carbon, oxygen and nitrogen and Fig. 5a shows the presence of nickel, carbon, and oxygen. In Figs. 4b and 5b display two edge splits attributed to the spin-orbital interaction in the Ni 2p spectrum. In Fig. 4b satellite peak at 856.4 eV, and the major peak of 2p3/2 is at 861.9 eV. And the confirmation of NiO were been indicated by at the peak 870.3 eV and the corresponding peak were held at 874.51 eV. The satellite is at 861.1 eV, and the major peak of 2p3/2 is precisely at 854.2 eV. The presence of NiO is indicated by the primary peak for 2p1/2 at 871.4 eV and its corresponding satellite peak at 879.7 eV. Figure 4c of C 1 s convoluted spectra of the NiO/g-C_3_N_4_ composite, peaks are at 284.5 eV(C-OH bond), 285.9 eV(C = C bond), 286.2(C-O-C bond), 288.5 (-COOH bond), and 289.3 (C-N bond) coincide with sp^2^ carbon components (C-C bond), (C-OH) are absorbed water molecule, and (C = OH bond) it is the components of ketone and carboxyl group fuctionalized.N1s spectrum Fig. 4d shows NiO/g-C_3_N_4_ composite was successfully synthesized, as indicated by the C-N peaks. The tertiary nitrogen N-(C)3 group has a binding energy of 400.5 eV, while the primary N1s peak, which has a binding energy of 398.8 eV, is caused by a sp2-hybridized aromatic N bond to a C atom (C = N-C). The peaks at 404.4 eV were attributed to the C-N-H group. For most prominent oxide peaks was shown at 530.5 eV which owing to O1s core levels in Fig. 4e and its shows the O2^−^ corresponding to Ni-O bond in the hematite. Three peak centred at 531.4 eV, 532.2 eV, and 533.3 eV, correspond to C = O, C-O and C-O-C groups respectively.

For the NiO/rGO composite that confirms the presence of the reduced graphene oxide. In the Fig. 5c shows the C 1 s spectra of provides the deconvoluted into several peaks at 284.8 eV, 285.7 eV, 286.5 eV, and 288.7 eV which is respective to the bond of (C-OH), (C-C), (C-O) and (O-C-O). For O1s attributed the peaks at 529.7 eV shown in the Fig. 5d. Followed by its peak centred at 531 eV, 531.6 eV, and 533 eV. Which corresponded to provide the bond (C-O), (Ni-O), (C = O), and (C-O-C) groups^39,40^.

**Fig. 6:**
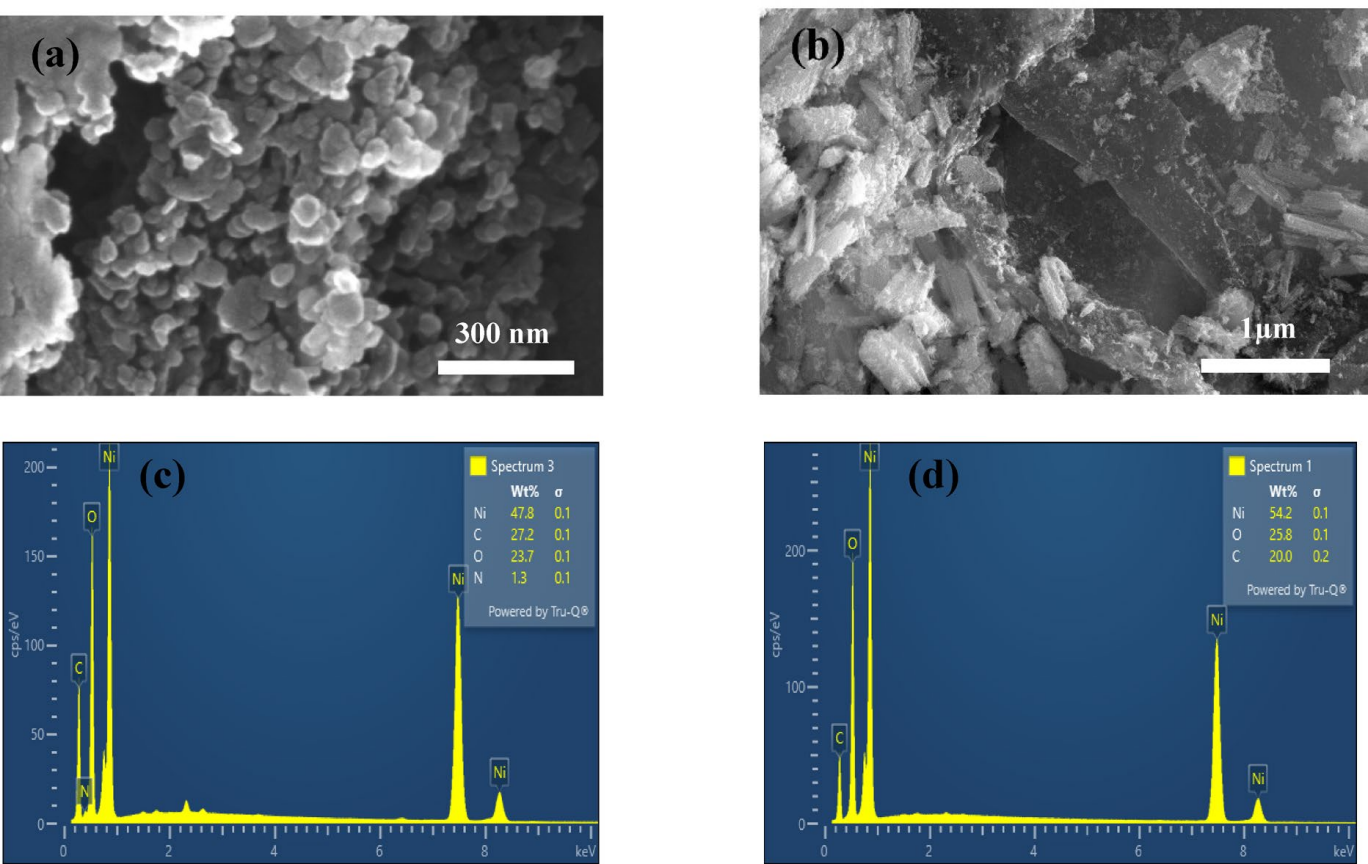
NiO/g-C_3_N_4_ and NiO/rGO (**a**, **b**) FESEM images, (**c**, **d**) EDAX analysis.

**Fig. 7:**
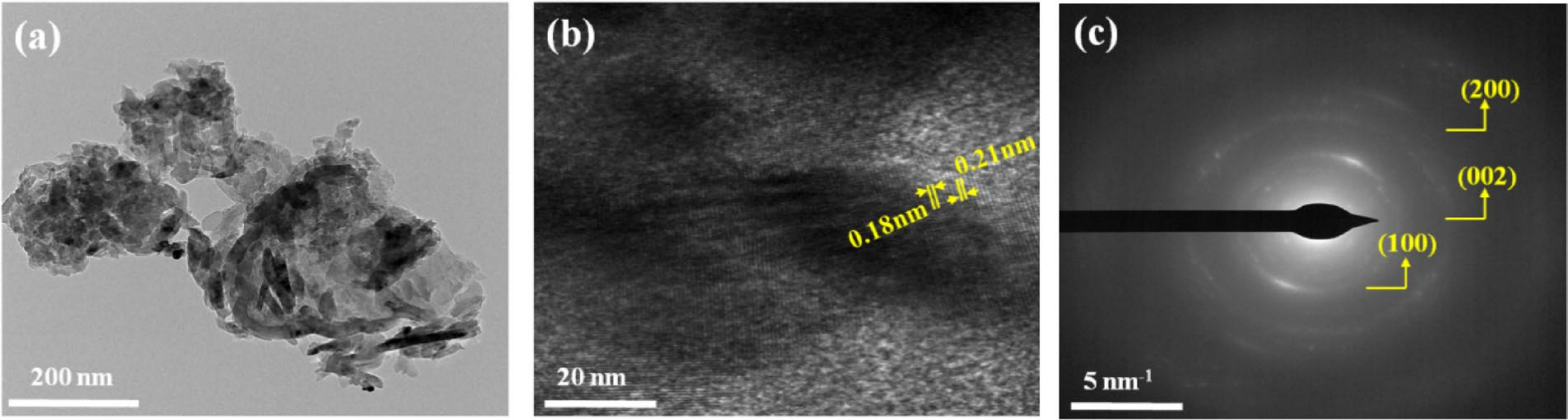
(**a**) TEM image, (**b**) HRTEM image, (**c**) SAED pattern for NiO/rGO.

As prepared NiO/g-C_3_N_4_ sample reveals nanoparticles for NiO with particle size 21 nm and sheet like structure of g-C_3_N_4_ Fig. 6a NiO/rGO samples shows nanorods like structures with length of 130 nm and diameter of 42 nm and densely arranged sheets are seen in Fig. 6b confirms the presence of rGO in NiO. Figure 6c, d displays the EDS scale for the prepared NiO/g-C_3_N_4_ and NiO/rGO, no other peaks are presents in EDS spectrum that exhibits the purity of composite. NiO/rGO displays rods are encapsulated by layer sheet of g-C_3_N_4_ with length and diameter of 161 and 21 nm are given in Fig. 7a HRTEM analyses reveals the pattern fringes spacing value of 0.18 nm and 0.21 nm and this is coordinated with *d* spacing value obtained from XRD Fig. 7b SAED pattern Fig. 7c confirms the polycrystalline nature of NiO and it gives diffraction rings that are ordered to the (100), (002), (200) planes of rGO and NiO.

**Fig. 8:**
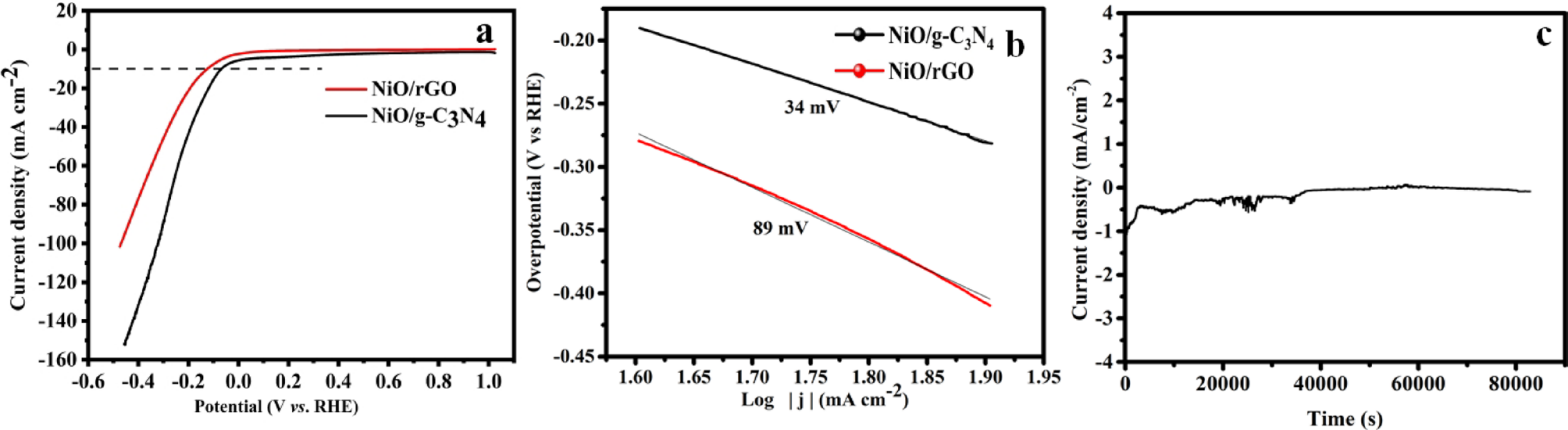
(**a**) LSV curve (**b**) Tafel plots (**c**) chronoamperometry curve at 10 mA cm^−2^ current density for 23 h.

To evaluate the HER performance of NiO/g-C_3_N_4_ and NiO/rGO catalyst, electrochemical experiment was performed using 3-electrode system in a 1 M KOH. Figure 8a displays the LSV curves of HER with an over potential of 126 mV and 73 mV at current density of 10 mA cm^−2^ for NiO/rGO and NiO/g-C_3_N_4_ electrode. NiO with g-C_3_N_4_ made immediate contact, which imporved the better electron transport and current response and is answerable for the low overpotential of NiO/g-C_3_N_4_. There are two mechanisms underlying HER kinetics: (1) here, H^+^ ions are absorbed on the electrode surface in the Volmer-Tafel process, where the absorbed H atoms are merged jointly to create H_2_ in the Volmer-Heyrovsky process, absorbed H atoms can be straightly bonded with H + ions to produce H_2_ through electron transfer. To determine the HER kinetics of the electrocatalyst, we have evaluated the Tafel slope value for the prepared catalyst, in this NiO/rGO catalyst shows 89 mV dec^−1^ and NiO/g-C_3_N_4_ catalyst shows 34 mV dec^−1^, respectively are given in Fig. 8b The lower Tafel slope of NiO/g-C₃N₄ confirms its superior HER catalytic efficiency primarily due to better charge separation and active site effectiveness, whereas NiO/rGO’s higher slope reflects relatively slower hydrogen adsorption or intermediate steps despite higher conductivity. The small tafel slope is indicative of the material’s rapid HER kinetics and is anticipated to align with the Volmer-tafel hydrogen formation mechanism. The performance is compared with some other reported works. g-C_3_N_4_@NiO (83 mV dec^−1^)^41^, Cu_2_O/g-C_3_N_4_ (55 mV dec^−1^)^42^, Co/g-C_3_N_4_ (44.2 mV dec^−1^)^43^, Ni/NiO@rGO (63 mV dec^−1^)^44^, Ni/NiO doped N doped AC (121 mV dec^−1^)^45^, g-C_3_N_4_/CeO_2_/Fe_3_O_4_ (102 mV dec^−1^)^46^. Figure 8c reveals the chronoamperometry studies at the constant current density of 10 mA cm^−2^ for 23 h. There was no noticeable degradation observed in the activity, its shows the excellent cyclic performance.

**Fig. 9:**
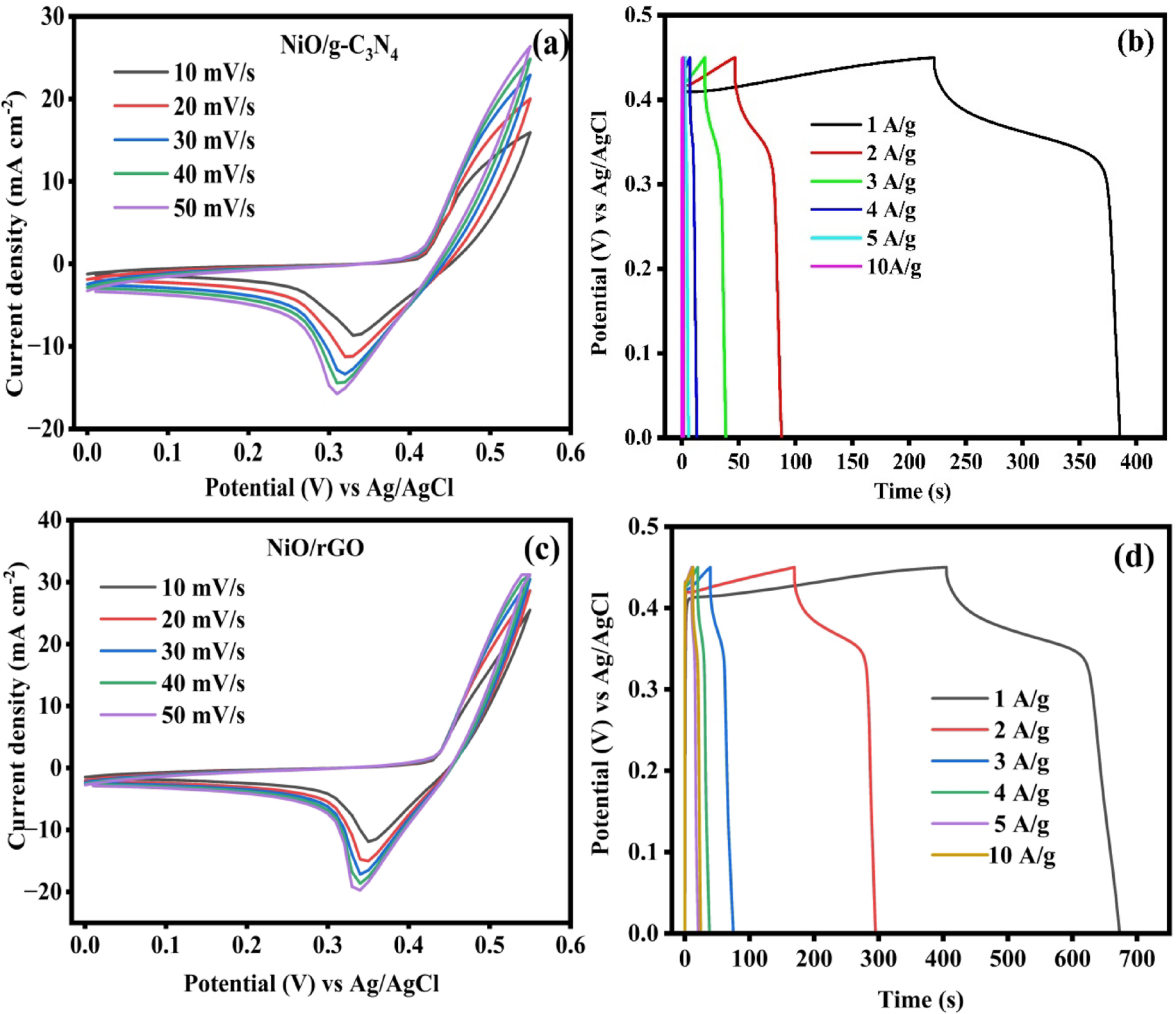
(**a**, **c**) CV curves of the NiO/g-C_3_N_4_ and NiO/rGO electrode for different sweep rates, (**b**, **d**) GCD curve of the NiO/g-C_3_N_4_ and NiO/rGO for different current densities.

Electrochemical performances of prepared NiO/g-C_3_N_4_ and NiO/rGO electrodes were further observed by CV and GCD. Figure 9a, c reveals the CV plot under various scan sweep between 10 and 50 mV/s at the potential of 0 to 0.6 V in 3 M of KOH. It indicates the pseudo capacitive behaviour reveals the redox reaction of Ni^2+^/Ni^3^. When the scan rate increases, the redox potential also increases in the electrode. The observed shift can be attributed to higher internal resistance and slower charge transfer at the electrode–electrolyte interface. As the scan rate increases, ion diffusion into the electrode bulk becomes limited, resulting in a response that is increasingly governed by surface processes. The charge and discharge of the prepared electrode were carried out from 1 to 10 A g current densities at the potential of 0–0.6 V are given in Fig. 9b, d. The non-linear GCD curves of the prepared electrode demonstrated the pseudo-capacitive faradic behavior in Ni^2+^/Ni^3+^ electronic states. The possible electrochemical reaction as written as.

$$NiO+OH^-\Rightarrow NiOOH+e^-$$

In an alkaline aqueous solution, the charge-discharge mechanism relies on the quasi-reversible faradaic redox process of Ni^2+^ to Ni^3+^ between the electrode/electrolyte interfaces. When NiO particles and KOH electrolyte undergo electrochemical redox, the surface Faradaic reaction of Ni^2+^ to Ni^3+^ takes place at the surface of NiO, whereas the reverse Faradaic reaction of Ni^3+^ to Ni^2+^ takes place in reduction^47^. The production of NiOOH can also increase electronic conductivity, which is a more effective way to increase the response of the capacitive current. NiO/r-GO electrode materials generated the highest responses in current and time for both the CV and GCD curves due to their increased electrical conductivity, increased surface area, decreased resistance, and synergistic effect between NiO/g-C_3_N_4_ and NiO/rGO composites^48^.

**Fig. 10:**
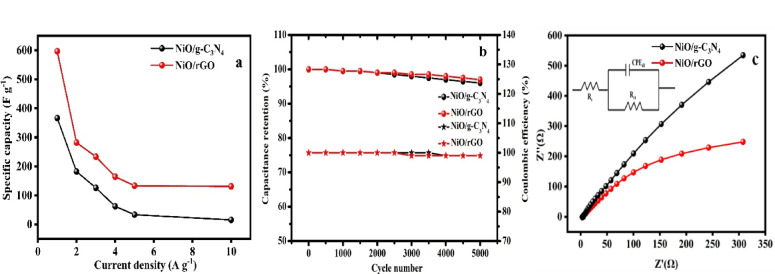
(**a**) specific capacitance at various current densities, (**b**) cyclic stability and (**c**) Nyquist plot (inset) fitted equivalent circuit of NiO/rGO and NiO/g-C_3_N_4_.

Figure 10a reveals the determined specific capacity of the prepared NiO/g-C_3_N_4_ and NiO/rGO electrodes are 366.6, 182.2, 126, 62.2, 33.3, 25 F g^−1^ for NiO/g-C_3_N_4_ and 597, 282, 233.3, 164.2, 133.7 and 131.8 F g^−1^ for NiO/rGO at the current density of 1, 2, 3, 4, 5 and 10 A g^−1^. NiO/rGO electrode possesses a high specified capacitance of 597 F g^−1^ compared to NiO/g-C_3_N_4_ it has 366 F g^−1^ at the current density of 1 A g^−1^. While specified capacitance decreases while increasing the current densities. The cyclic stability of the electrodes was achieved by GCD with a cycle number of 5000 at the current density of 10 A g^−1^. The retention of the prepared electrodes is 96 and 97% with a coulombic efficiency of 99% for NiO/g-C_3_N_4_ and NiO/rGO is given in Fig. 10b The Nyquist plot of both samples, which shows the relationship between the complex impedance’s real and imaginary parts are revealed in Fig. 10c and the fitted equivalent circuit for Nyquist plot is given in inset of Fig. 10c. It is clear that although the slope of both curves varies, the plot does not exhibit a discernible semi-circular shape, especially in the region with lower impedance values. Both samples are suitable for capacitor applications because of their higher capacitance, as indicated by the plot’s straight line.

**Fig. 11:**
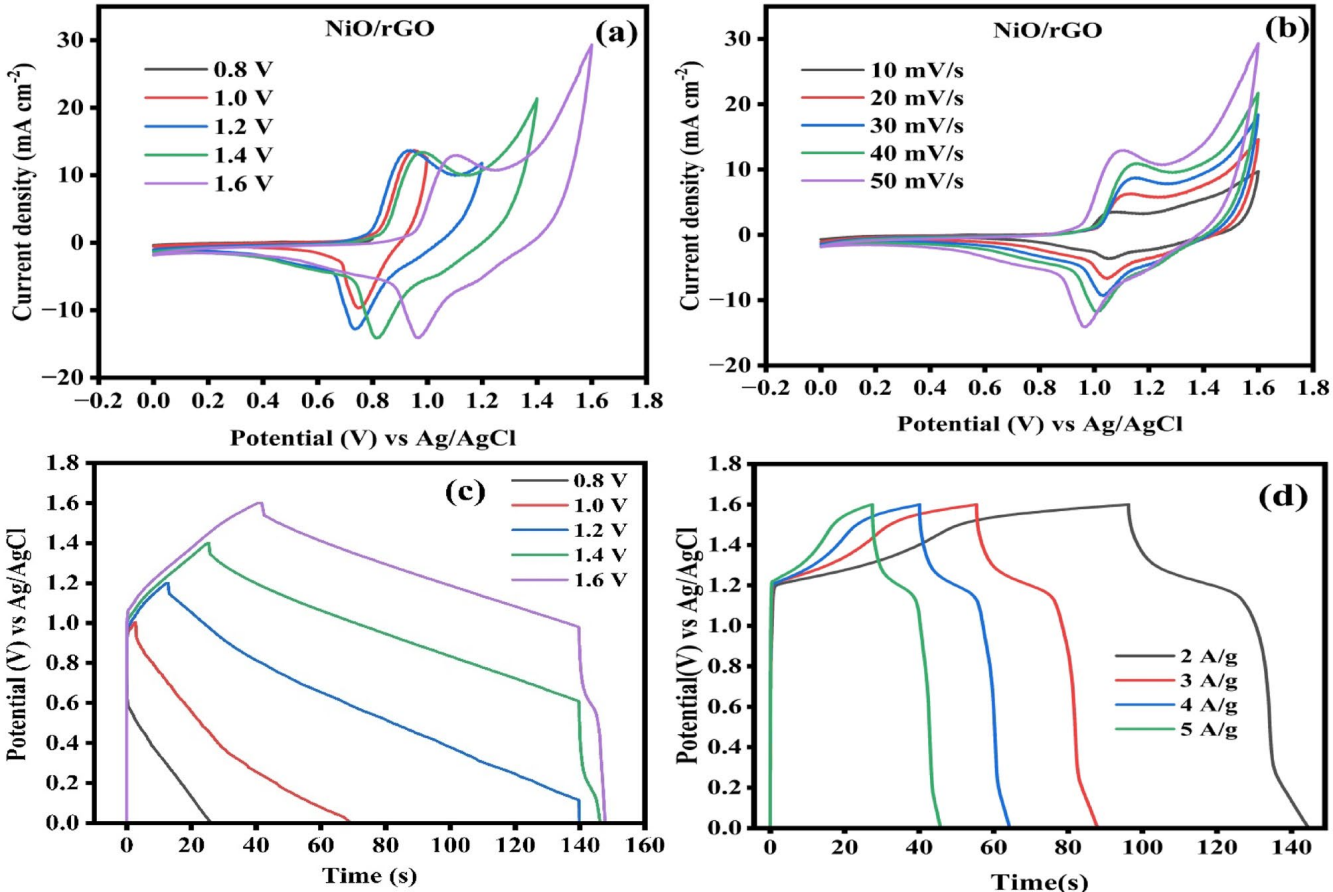
(**a**) CV curve at various potentials, (**b**) CV curve at various scan sweeps, (**c**) GCD curve at various potentials and (**d**) GCD curve at various current densities for the fabricated NiO/rGO//AC devices.

**Fig. 12:**
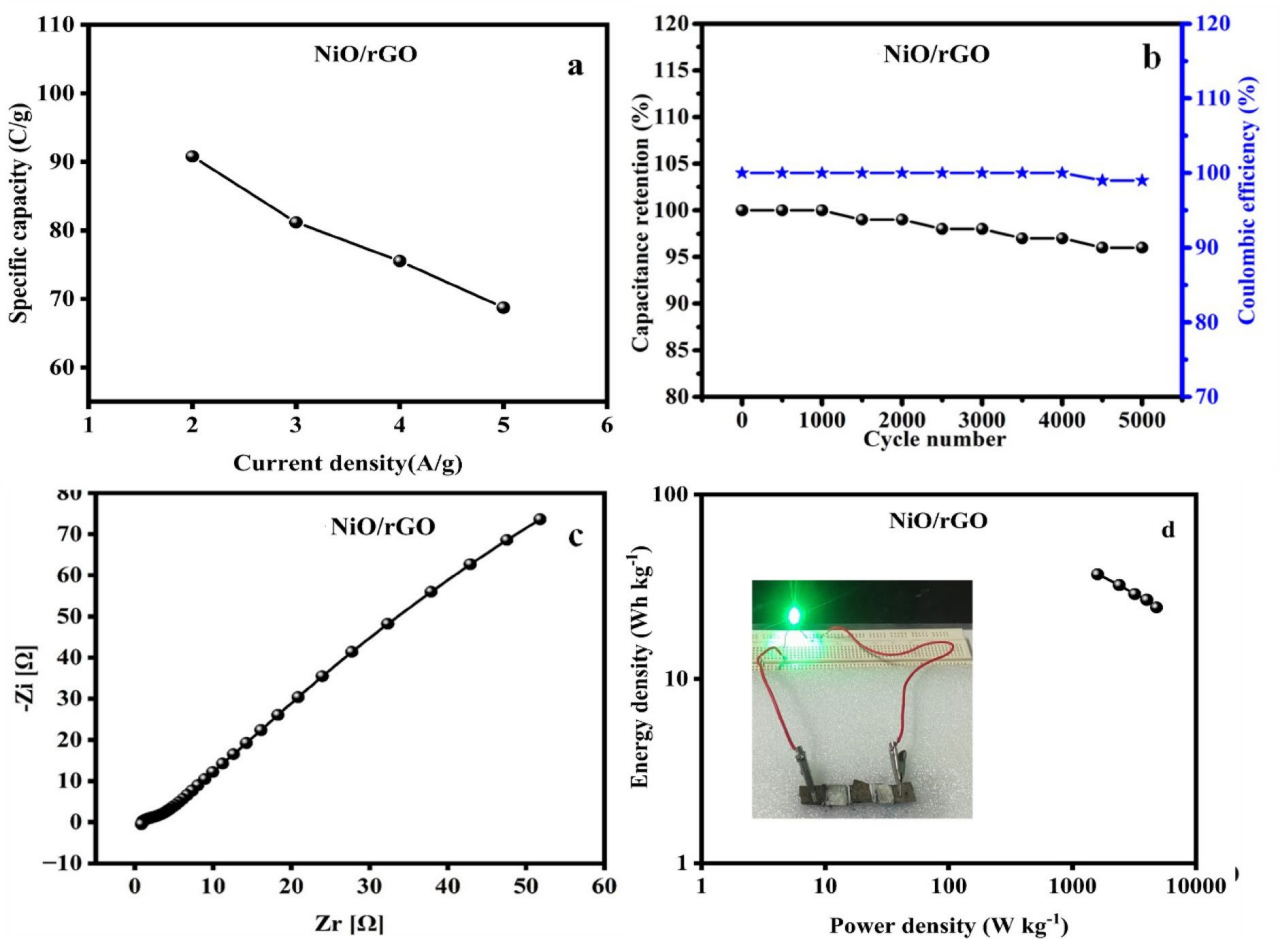
(**a**) specific capacitance vs. current densities, (**b**) cyclic stability, (**c**) Nyquist plot and (**d**) Ragone plot for the fabricated NiO/rGO//AC devices.

CV and GCD methods were used to estimate the NiO/rGO//AC ASC device. The CV plot obtained at different potentials from 0.8 to 1.6 V at the scan speed of 50 mV/s and scan speed varied from 10 to 50 mV/s for the range of potential 0 to 1.6 V are shown in Fig. 11a, b. It displays a pair of redox peaks in a quasi-triangular shape, the combined effect of electric double-layer capacitance (EDLC) and pseudo-capacitance is evident from this. Figure 11c, d displays the GCD plot for different potentials from 0.8 to 1.6 V at the current density of 2 A g^−1^, and current densities varied from 1 to 5 A ^-1^g^−1^ with the potential range from 0 to 1.6 V. GCD curves exhibit a plateau at different current densities, suggesting that the electrodes continue to function in a highly reversible manner consistent with their redox processes. The evaluated specific capacitances for the fabricated devices are 90.8, 81.17, 75.53, and 68.73 F g^−1^ at the current density of 2, 3, 4 and 5 A g^−1^ are given in Fig. 12a Here, the capacitance values are reduced with increasing the current densities. The prepared ASC electrode displays a capacitance retention of 97% with a cyclic stability of 99% even up to 5000 cycles at the current density of 10 A g^−1^ Fig. 12b. This indicates excellent electrochemical repeatability and long-term cyclic stability.

Figure 12c shows the results of an EIS conducted to examine the charge transfer and ion diffusion mechanism for the manufactured ASC NiO/rGO//AC device in the 0.1 Hz to 100 MHz range. The device reveals the R_s_ and R_ct_ values are 0.5 Ω and 2.4 Ω, respectively. According to the Nyquist diagrams, the electrode shows the optimal double-layer capacitive behaviour in the low-frequency region, as indicated by the imaginary part of the impedance spectra and the vertical increase in the y-axis. The low electron transfer internal resistance in the films is also indicated by the extremely tiny semicircles seen in the high-frequency region of the Nyquist plots^49^. Information regarding the effectiveness of electron transfer at the electrode-electrolyte interface can be found in the diameter of the semicircle that forms. The x and y axis represent the energy and power levels in the Ragone plot. The material connected to the series glows the green LED by AC. 9 V batteries serve to charge the devices for one minute. Over duration of 135 s, the green LED remains on, gently varied in brightness are shown in Fig. 12d (inset).

The electrochemical performance of the synthesized NiO/rGO electrode was systematically compared with previously reported metal oxide-, rGO-, and g-C₃N₄-based composites (Table 1). The present electrode delivered a remarkable specific capacitance of 597 F g⁻¹ at 1 A g⁻¹, which is significantly higher than those of other NiO/rGO systems such as 192 F g⁻¹ at 0.2 A g⁻¹^35^ and 171.3 F g⁻¹ at 0.5 A g⁻¹^59^, as well as pristine NiO (270 F g⁻¹) and NiO@rGO (395 F g⁻¹)^22^. It also surpasses g-C₃N₄/NiO (207.8 F g⁻¹)^60^ and g-C₃N₄-based hybrids such as Co/GCN-NS (458 F g⁻¹) and Cu/GCN-NS (154 F g⁻¹)^57^. Such enhancement can be ascribed to the synergistic interaction between NiO and rGO, where the conductive rGO matrix facilitates rapid charge transfer and the intimate interfacial contact provides abundant electroactive sites. Beyond capacitance, the fabricated asymmetric device revealed a maximum energy density of 36.88 W h kg⁻¹ at a power density of 1599.61 W kg⁻¹, while still retaining a high power output of 4798.03 W kg⁻¹ at 24.43 W h kg⁻¹. These values are superior to those of several reported transition metal oxide–carbon composites, including NiO/rGO//AC (31.42 W h kg⁻¹)^50^, g-C₃N₄/Co₃O₄ (27.9 W h kg⁻¹)^52^, Ni(OH)₂/g-C₃N₄/rGO (33.38 W h kg⁻¹)^53^, and Cu₂O–CuO/rGO (17.9 W h kg⁻¹)^54^, and far exceed MnO₂-based systems such as MnO₂@BL (11.47 W h kg⁻¹)^55^ and MnO₂/CNT (27 W h kg⁻¹)^56^. Even compared to NiO–NiO/g-C₃N₄, which delivered only 7.91 W h kg⁻¹^51^, the improvement is substantial. Furthermore, the NiO/rGO electrode retained 99% of its capacitance after extended cycling, demonstrating excellent durability that clearly outperforms previously reported NiO/rGO electrodes (72.1%^35^, 79%^59^ and g-C₃N₄/NiO (70%^60^). This outstanding retention underscores the structural robustness imparted by the rGO matrix, which effectively accommodates volume changes during cycling and prevents electrode pulverization. Overall, the present NiO/rGO system demonstrates a compelling combination of high capacitance, energy density, power density, and cycling stability, thereby positioning itself as a superior electrode material for next-generation supercapacitors.

**Table 1:** Comparison study of metal oxide composite based supercapacitor device with previous report.

S.no	Electrode material	Electrolyte	Specified capacitance (F g^−1^)	Current density (A g^−1^)	Energy density (W h kg^−1^)	Capacitance retained (%)	Ref papers
**1.**	i) Co/GCN-NS ii) Cu/GCN-NS	KOH	458 154	0.5 0.5	- -	94 (5000 Cycles)	^57^
**2.**	Fe, Cu, and Zn (SnO2) doped	KOH	Fe-270 Cu-251 Zn-223	0.5 0.5 0.5	- - -	98 (2000 Cycles)	^58^
**3.**	NiO/GCN	KOH	338.68	1	10.13	96 (5000 Cycles)	^21^
**4.**	i) NiO ii) NiO@rGO	KOH	270 395	1 1	10.2 17.5	- -	^22^
**5.**	NiO/rGO	KOH	192	0.2	-	72.1 (2000 Cycles)	^35^
**6.**	NiO/rGO	KOH	171.3	0.5	-	79 (2000 Cycles)	^59^
**7.**	g-C_3_N_4_/NiO	KOH	207.8	1	-	70 (1000 Cycles)	^60^
**8.**	NiO/rGO	KOH	597	1	36.88	97 (5000 Cycles)	This work

### Structural study after stability

**Fig. 13:**
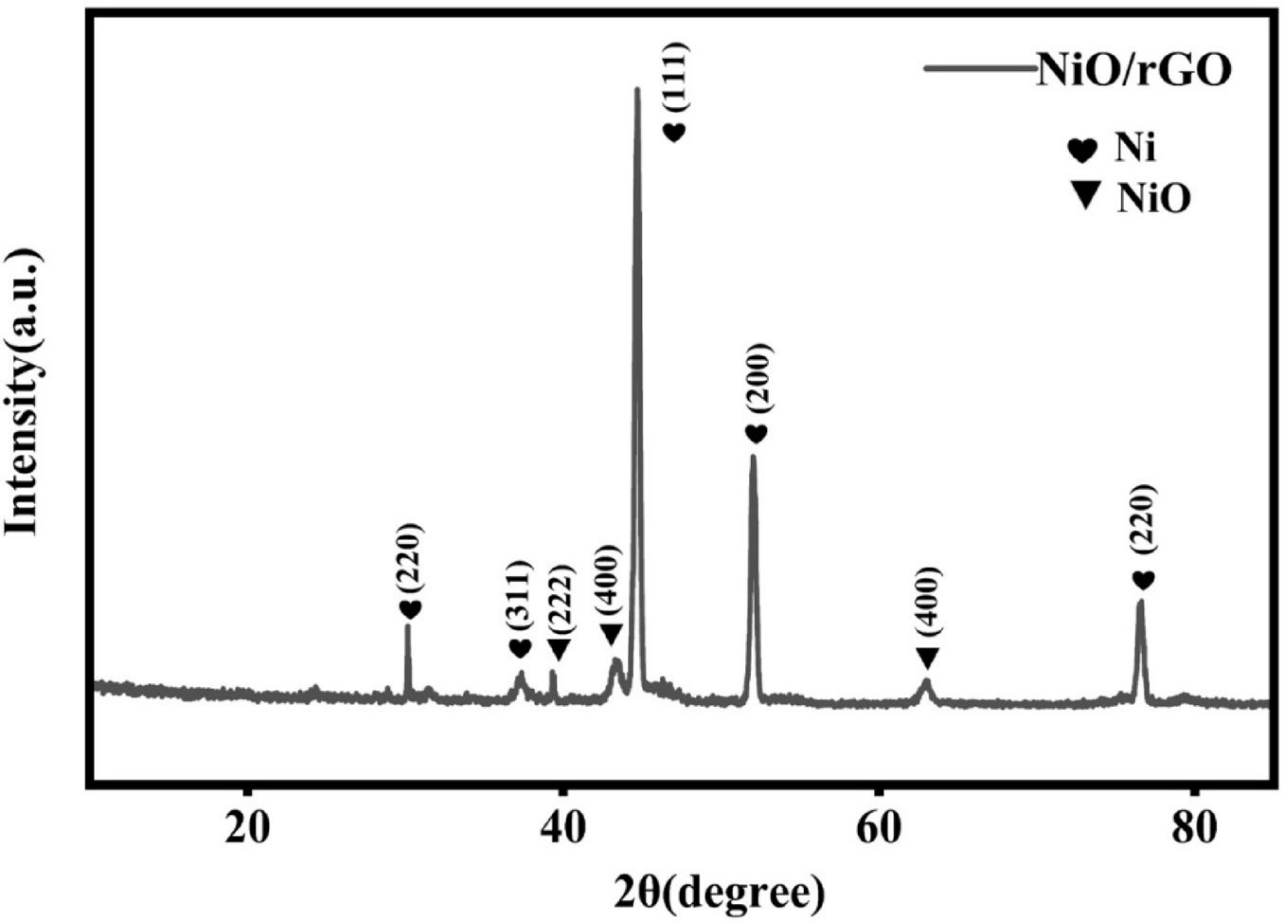
XRD image for NiO/rGO after cyclic stability.

In Fig. 13 NiO shows that prominent diffraction peaks at 2θ values of 30.19◦, 37.36◦, 39.30◦, 44.68◦, 62.96◦, and 76.62◦, which correspond to the (220), (311), (222), (400), (111), (200), (400), and (220) planes, respectively. These planes correspond to the face-centered cubic (FCC) structure of NiO. The post test results that the presence of NiO shows no deterioration after the electrochemical studies.

## Conclusion

In summary, we synthesized two novel composite materials, NiO/rGO (nickel oxide/reduced graphene oxide) and NiO/g-C₃N₄ (nickel oxide/graphitic carbon nitride), for dual applications in supercapacitors and electrochemical water splitting. The composites were synthesized through a simple and efficient approach, leveraging the synergistic effects between NiO nanoparticles and conductive substrates (reduced graphene oxide and graphitic carbon nitride) to enhance both the electrochemical performance and catalytic activity. The NiO/rGO composite exhibits superior electrochemical capacitance, high cycling stability as well as elevated rate capabilities, demonstrating its potential as a supercapacitor electrode material. Meanwhile, the NiO/g-C₃N₄ composite shows remarkable catalytic performance in hydrogen evolution reaction (HER), highlighting its applicability for electrochemical water splitting.The combined advantages of these composites in energy storage and conversion systems underscore their versatility and potential for sustainable energy applications. Our findings offer a promising pathway for the development of advanced materials for next-generation energy devices.

## Data Availability

All data generated or analysed during this study are included in this published article.
